# Low adoption in women’s professional football: teams that used the Nordic Hamstring Exercise in the team training had fewer match hamstring injuries

**DOI:** 10.1136/bmjsem-2022-001523

**Published:** 2023-05-05

**Authors:** Jan Ekstrand, Anna Hallén, Håkan Gauffin, Håkan Bengtsson

**Affiliations:** 1 Department of Health, Medicine and Caring Sciences, Linköping University, Linkoping, Sweden; 2 Football Research Group, Linköping, Sweden; 3 Department of Orthopaedics Department of Biomedical and Clinical Sciences, Linköping University, Linkoping, Sweden

**Keywords:** training, elite performance, epidemiology, muscle damage/injuries, rehabilitation

## Abstract

**Objectives:**

The primary objective was to study the reach, effectiveness, adoption, implementation and maintenance of the Nordic Hamstring Exercise (NHE) programme in women’s elite teams in Europe in the 2020–21 season. The secondary objective was to compare hamstring injury rates between teams that used the NHE programme regularly in team training and teams that did not.

**Methods:**

Eleven teams participating in the Women’s Elite Club Injury Study during the 2020–21 season provided data about injury rates and the implementation of the NHE programme.

**Results:**

One team (9%) used the full original NHE programme, and four teams used the programme in the team training during parts of the season (team training group, n=5). Five teams did not use the NHE, or used it only sporadically for individual players, and one team used NHE only for players with a previous or current hamstring injury (no team training group, n=6). The team training group had a lower incidence of hamstring injuries during match-play (1.4 vs 4.0, p=0.028) than the non-team training group while no difference between groups was shown for the hamstring injury incidence in training (0.6 vs 0.7, p=0.502).

**Conclusion:**

A low adoption of the NHE programme was reported during the 2020–21 season. However, teams that used NHE for the whole team or most players had a lower hamstring injury incidence at match-play than teams that did not use the NHE or used it for individual players only.

**WHAT IS ALREADY KNOWN ON THIS TOPIC**

- Hamstring injury is the most common injury subtype in women’s professional football players.
- The number of acute and recurrent hamstring injuries in football could be reduced by the Nordic Hamstring Exercise (NHE) programme.
- Previous studies have reported a low adoption of the NHE programme in men’s professional football in Europe.

**WHAT THIS STUDY ADDS**

- Medical staff in this cohort had a generally positive attitude towards the NHE.
- Few teams used the NHE programme during the 2020–21 season.
- The match hamstring injury incidence and recurrence rate was lower in teams that used the NHE in their team training for all or most players compared with teams that did not.
- The majority of the teams reported that they would continue to use the NHE in the future, but no team said that it would be their only hamstring prevention measure.

**HOW THIS STUDY MIGHT AFFECT RESEARCH, PRACTICE OR POLICY**

- Our findings may increase the use of the NHE since teams that used the exercise for most of their players was shown to sustain fewer hamstring injuries and had on average 50 less hamstring injury absence days during the season compared with teams that did not use the NHE to the same extent.
- The potential to reduce hamstring injuries is an important point to convince coaching staff to include the NHE in team training and not only for a selection of players with previous hamstring injuries.

## Introduction

Hamstring injury appear to be the most^1 2^ or second most common^3^ injury subtype in women’s elite football players constituting 12%–16% of all time-loss injuries.^1–3^ A team with a 19–22 players squad could thus typically expect three to four hamstring injuries each season.^1 3^

In women’s football, the Nordic Hamstring Exercise (NHE) programme has been reported to reduce acute hamstring injuries by 80%.^4^ The corresponding reduction in men’s football has been reported at 50%–70%.^5–8^

The NHE programme has, however, not been widely adopted in professional football in Europe. According to a study a from 2015, only 13% of included teams reported that they used the complete or parts of the NHE programme,^9^ indicating a low adoption and implementation of the programme. To our knowledge, no formal evaluation of the implementation of the NHE in women’s elite football has been done.

The primary objective of this study was to study the reach, effectiveness, adoption, implementation and maintenance (RE-AIM) of the NHE programme in Women’ Elite Club Injury Study (WECIS) teams that participated in the UEFA Women’s Champions League in the 2020–21 season.

The secondary objective was to compare the incidence and burden of hamstring injuries between teams that used the full or modified NHE programme in the team training during the 2020–21 season and teams that did not use the NHE or used it only for players with previous hamstring injuries or in the rehabilitation of hamstring injuries.

## Materials and methods

In this observational cohort study, injury data were collected prospectively while questionnaire data were collected retrospectively.

### Background of the study

The study was carried out by Football Research Group (FRG) on behalf of UEFA. FRG is an international research team conducting studies on football injuries. On behalf of and in cooperation with UEFA, FRG has carried out a men’s UEFA Elite Club Injury Study (ECIS) continuously since 2001.^10–13^ During the 2017–18 season, FRG started a pilot study on women’s elite clubs in Europe, using the same validated methodology as in the ECIS.^14–16^ Thirteen teams participated in the pilot study, 12 of them participating in the UEFA Women’s Champions League and 11 of these 12 qualified for the round of 32 that season. Based on the positive results from the pilot study, UEFA decided to initiate and fund a continuous WECIS starting from season 2018–19. For continuity reasons, UEFA has decided that teams that participated in the pilot study should be invited to continue participating.

### Inclusion criteria for study participants

This is a substudy of the WECIS carried out during the 2020–21 season. We only included teams that sent us full data for the entire season, adding up to 11 teams (including a total of 288 players) from 7 countries (Chelsea FC and Manchester City from England, FC Barcelona and Club Athlético Madrid from Spain, AFC Ajax and PSV Eindhoven from the Netherlands, Juventus FC and AC Milan from Italy, Olympique Lyonnais from France, Fc Bayer München from Germany and Sporting Club de Portugal from Portugal). All 11 teams delivered complete data for the whole season and provided answers to the questionnaire.

### Exposure and injury data collection

The study period covered the full 2020–21 season, starting in July 2020 and finishing in May 2021. The methodology of the injury data collection procedure has been described in detail in previous publications.^14–16^ On inclusion, all participating team assigned a member of their medical staff to act as contact person and to be responsible for all data collection during their study participation. In most cases, the first team physician or physical therapist was assigned as contact person. Contact persons were provided with a study manual to inform them about the data collection procedure and all operational definitions used in the study (table 1). Exposure and injury data were sent to the study group monthly. All data were reviewed by members of the study group to make sure that it was complete and in agreement with the study methodology. Contact persons were asked to complete their reports if any missing or erroneous data were identified during the review process.

**Table 1:** Operational definitions

Training session	Team training that involved physical activity under the supervision of the coaching staff
Match	Competitive or friendly match against another team.
Injury	Any physical complaint sustained by a player that resulted from a football match or football training and led to the player being unable to take full part in future football training or match play.
Hamstring injury	An acute onset distraction injury or gradual onset injury to the hamstring muscle group.
Recurrent injury	Injury of the same type and at the same site as an index injury occurring previously during the same season.
Early recurrence	Recurrent injury that occurs within 2 months after return to full participation from the index injury.
Injury incidence	Number of injuries per 1000 player hours ((Σ injuries/Σ exposure hours)×1000).
Injury burden	Number of lay-off days per 1000 player hours ((Σ lay-off days/Σ exposure hours)×1000).

### The survey questionnaire

The survey was conducted using a questionnaire addressing key issues related to the RE-AIM of the NHE programme.^17^ In addition to the questions that were intended to evaluate the RE-AIM of the NHE programme (box 1), the questionnaire also included a description of the NHE protocol with the initial programme (the 10-week progression model) and the weekly maintenance programme (one session each week).^18^

Box 1 **Survey questions (response options and reach, effectiveness, adoption, implementation and maintenance framework domains are shown in parenthesis)**

- Are you familiar with the Nordic Hamstring Exercise programme aimed at reducing hamstring injuries? (Yes; No) (Reach)
- Have you used the complete original Nordic Hamstring Exercise programme (the 10-week progression as suggested by Mjølsnes *et al*^18^) in your first team squad at the start of season 2020–21? Choose one option: (Yes, the complete 10-week programme; Yes, but only for 7–9 weeks; Yes, but only for 5–6 weeks; Yes, but only for 4 weeks or less; No, not at all) (Adoption)
- With which players in your first team squad, did you use the original Nordic Hamstring Exercise programme during the 2020–21 season? (All players from the first team squad; Players with a history of hamstring injury only; No players; Other selection criteria (if yes, please describe your selection criteria)) (Adoption)
- How many players in your first team squad completed the initial Nordic Hamstring Exercise programme (the complete 10-week programme) during the season 2020–21? (>75% of players; 50%–74%; 25%–49%; <25%) (Implementation)
- Have you used a weekly maintenance programme with the Nordic Hamstring Exercise programme (one session each week as suggested by Petersen *et al*^5^) in your first team squad during season 2020–21? Choose one option: (Yes, every week; Yes, most weeks; Yes, but sporadically; No, not at all) (Adoption)
- With which players in your first team squad did you use the weekly maintenance programme during the 2020–21 season? Choose one option: (All players from the first team squad; Players with a history of hamstring injury only; No players; Other selection criteria (if yes, please describe your selection criteria)) (Adoption)
- How many players in your first team squad completed the weekly maintenance programme during the 2020–21 season? Choose one option: (>75% of players; 50%–74%; 25%–49%; <25%) (Implementation)
- Have you used any other (modified) Nordic Hamstring Exercise protocol with your first team squad at the start of the 2020–2021 season? (No; Yes, please describe the protocol used in your team) (Adoption)*
- Have you used the previously described modified Nordic Hamstring Exercise protocol in your first team squad during the competitive 2020–21 season? Choose one option: (Yes, every week; Yes, most weeks; Yes, but sporadically; No, not at all) (Adoption)*
- With which players in your first team squad did you use the described modified Nordic Hamstring Exercise protocol during the 2020–21 competitive season? Choose one option: (All players from the first team squad; Players with a history of hamstring injury only; No players; Other selection criteria (if yes, please describe your selection criteria)) (Adoption)*
- How many players in your first team squad completed the previously described modified Nordic Hamstring Exercise protocol during the 2020–21 season? Choose one option: (>75% of players; 50%–74%; 25%–49%; <25%) (Implementation)*
- Have you experienced any complaints about the Nordic Hamstring Exercise programme from players in your first team squad during the 2020–21 season? Choose one option: (Many; More than a few; A few; No complaints) (Effectiveness)
- How satisfied are you with the Nordic Hamstring Exercise programme in your first team squad? (Very dissatisfied; Dissatisfied; Indifferent; Satisfied; Very satisfied) (Effectiveness)
- With regard to the Nordic Hamstring Exercise programme, please let us know how you agree or disagree with each of the following statements: (1) it reduces injuries; (2) it makes more players available for team selection; (3) players can return to play sooner after injury; (4) it reduces re-injuries; (5) it is really easy to get players to do the programme; (6) the players really like the programme and see its value; (7) it causes muscle soreness in players; (8) it increases sprint speed and acceleration; (9) it increases hamstring muscle strength. (Fully agree; Partly agree; Indifferent; Partly disagree; Fully disagree) (Effectiveness)
- Do you intend to use the Nordic Hamstring Exercise programme for your first team squad in the future? (Yes; No; We have not thought about it yet) (Maintenance)
- Which hamstring injury prevention strategy for your first team squad do you intend to use in the future? (Nordic Hamstring Exercise programme is the only strategy we will use in the future; Nordic Hamstring Exercise programme is part of, but not the only strategy we will use in the future; We will have a hamstring injury prevention strategy, but the Nordic Hamstring Exercise programme is NOT part of this; We will not have a hamstring injury prevention strategy in the future; We have not thought about it yet) (Maintenance)
- Did your first team squad use any specific exercises/exercise programmes other than the Nordic Hamstring Exercise programme to prevent hamstring injuries in the 2020–21 season? (Yes; No) If yes, please describe. (Adoption)

*Additional questions in the 2020–21 survey not included in the original 2012–14 survey.^9^

The survey aimed to evaluate if the NHE programme had been used, how it was used and if other preventive measures were used in conjunction or instead of the NHE in teams participating in WECIS during the 2020–21 season. The contact persons from these teams were informed about the survey through email. If contact persons agreed to participate, they were given access to the survey through an online survey software (SurveyMonkey, California, USA).

The questionnaire was distributed in December 2021 and automatic reminders were sent after 3, 7 and 10 days.

### Patient and public involvement

This study was done without patient (player) involvement; patients (players) were not invited to comment on the study design or contribute to this document’s drafting.

### Data analyses

Teams were categorised in either a team training group or a non-team training group based on their responses to the questionnaire. The team training group included those teams that reported that they had used the NHE programme in their team training (including >75% of first team players) during the 2020–21 season. The non-team training group consisted of those teams that reported that they had not used the (full or modified) NHE programme during the 2020–21 season and teams that had used the programme only for a selection of their players (players with a previous hamstring injuries or players currently in rehabilitation following a hamstring injury).

Descriptive variables are presented with medians and IQRs due to the limited sample size. Injury incidence was calculated as the number of injuries per 1000 hours and described with a 95% CI. The injury burden was calculated as the number of lay-off days per 1000 hours. Comparisons of incidences were made using a rate ratio with 95% CI and tested for statistical significance with Poisson regressions using match exposure hours as an offset.

Analyses were two-sided, and the significance level was set at p<0.05.

## Results

### Reach

All teams (n=11) answered the questionnaire giving a response rate of 100%. In addition, all teams reported that they were familiar with the NHE programme indicating an excellent reach.

### Adoption and implementation

Five teams in the no-team training group reported that they had not used the NHE.

The team training group consisted of the only team (1/11=9%) that reported that they had used the complete NHE programme as intended, with an initial 10-week progression followed by a weekly maintenance programme during the rest of the season, and four teams that reported that they had used the programme in the team training during 5–9 weeks during the preseason period, during the competitive season or during both. In the no-team training group, one team reported that they used the programme for players with a history of hamstring injury or as part of the rehabilitation of new hamstring injuries while the other five teams did not use the NHE at all during the 2020–21 season.

### Satisfaction and complaints (effectiveness)

Out of the six teams that used the NHE, three teams (50%), all in the team training group, reported that they were satisfied or very satisfied with the NHE. Three teams (50%) reported that they were indifferent to the method.

The majority, four out of six (67%), of the teams that had used the NHE during the 2020–21 season reported that players had complained about the exercise during the season. Most of these teams, three out of four (75%), reported few complaints while the last team reported more than a few complaints. In most cases, these complaints were regarding muscle stiffness or soreness after training sessions.

### Future use of the NHE (maintenance)

Six clubs reported that they would include NHE as a part of their hamstring injury prevention strategy in the future, but none said that the NHE programme would be their only hamstring injury prevention measure. One team reported that they would not include the NHE in their future training while the remaining four teams reported that they had not yet decided whether they would include the NHE in their future training.

### Exposure and injury data

In total, 288 individual players reported 61 hamstring injuries during a total of 66 868 exposure hours. Thirty-nine injuries (64%) occurred during 58 592 training hours and 22 injuries (36%) during 8276 hours of match-play. Hamstring injuries accounted for 13% of all injuries (n=470) and caused 8% of the total number of absence days due to injuries. The risk of a hamstring injury was four times higher at match-play compared with training.

The team training group reported 20 hamstring injury occurrences (14 (70%) in training and 6 (30%) in matches) during a total of 28 424 exposure hours (24 143 training hours and 4281 match hours). In the no-team training group, 41 hamstring injury occurrences (25 in training and 16 in matches) were reported during a total of 38 444 exposure hours (34 449 training hours and 3995 match hours). Hamstring injury data and exposure hours, with comparisons between the team training and the non-team training groups, are presented in tables 2 and 3.

**Table 2:** Exposure and hamstring injury data in teams where all players performed the Nordic Hamstring Exercise (NHE) (n=5) and teams where only some players performed NHE (n=6)

	Team training (n=5) Median (IQR)	No team training (n=6)
Training exposure hours	3753 (3729–5425)	5609 (4900–5995)
Match exposure hours	829 (759–877)	694 (591–731)
Total exposure hours	4513 (4426–6543)	6328 (5641–6676)
Training hamstring injuries	2 (2–3)	5 (3–5)
Match hamstring injuries	1 (1–2)	3 (0–4)
Total hamstring injuries	4 (3–4)	7 (5–10)
Hamstring injury burden	10 (7–12)	16 (9–26)
Total lay-off days following training hamstring injuries during the 2020–21 season	13 (11–42)	49 (23–56)
Total lay-off days following match hamstring injuries during the 2020–21 season	31 (11–42)	35 (0–126)
Total lay-off days absence following all hamstring injuries during the 2020–21 season	76 (31–84)	90 (56–149)

Hamstring injury burden is expressed as the number of lay-off days per 1000 player hours.

**Table 3:** Comparison of hamstring injury incidences between teams in which all players performed the Nordic Hamstring Exercise (NHE) (n=5) and teams in which only some players performed NHE (n=6)

	Team training (n=5)	No team training (n=6)	Rate ratio	P value
Training hamstring injury incidence, injuries/1000 hours (95% CI)	0.6 (0.3 to 1.0)	0.7 (0.5 to 1.1)	0.80 (0.42 to 1.54)	0.502
Match hamstring injury incidence, injuries/1000 hours (95% CI)	1.4 (0.6 to 3.1)	4.0 (2.5 to 6.5)	0.35 (0.14 to 0.89)	0.028
Hamstring injury recurrence incidence, injuries/1000 hours (95% CI)	0.0 (0.0 to 0.2)	0.3 (0.2 to 0.5)	0.12 (0.02 to 0.95)	0.045

## Discussion

The current study showed excellent reach but low adoption and implementation of the full NHE programme among women’s elite teams. While adoption and implementation of the programme was low, respondents had good knowledge and a positive attitude towards the NHE. In addition, teams that used NHE for the whole team or most players had a lower hamstring injury incidence at match-play and hamstring injury recurrence incidence than teams that used NHE only for individual players or not at all. However, no difference in hamstring injury incidence in training was shown between teams that used NHE for the whole team or most players and teams that used NHE only for individual players or not at all. These findings were similar to previous reports in men’s elite football.^9 19^

### Why is it important to specifically study women’s teams and repeat studies previously performed in men’s teams?

Women are significantly under-represented in sport research,^20 21^ but still distinct differences have been shown in male and female injuries in football.^1 22 23^ There are different hormonal characteristics in women compared with men and use of contraceptives which could influence the injury rate of elite women’s football players.^21 24^ Men have 15-fold to 20-fold greater circulating testosterone than women which influence performance^25^ and possibly affects the injury panorama. Furthermore, differences in phycological factors have an impact.^26^ All this calls for separate studies for women elite athletes with sometimes different methodological considerations.^27^

### Is the NHE programme shown effective?

There are several well-designed controlled studies showing that the NHE programme effectively reduces injuries^4 5^ as well as systematic reviews and meta-analysis that reports a preventive effect.^6 7^ In addition, elite male teams that implemented NHE in team training and used it with most players have been shown to sustain fewer hamstring injuries than teams that only used the NHE for individual players with current or a history of a hamstring injury, a result that was replicated in this present study.^19^ Existing literature thus indicates that the NHE may be effective in reducing the number of hamstring injuries in football. The generally positive attitude towards the NHE that was reported in our survey indicates that health professionals in women’s professional football also believe that the exercise is indeed effective.

### Why is the NHE programme not in common regular use (adoption and implementation) in elite football despite these positive research findings?

The fact that the NHE programme is not widely used in elite women’s football may be considered surprising since the general attitude towards the programme in this study was shown to be positive (effectiveness) and since the scientific literature seem to indicate that the programme could efficiently reduce the number of hamstring injuries in football. Especially given the fact that hamstring injury is one of the main contributors to injury absence in professional football. However, the surprise may be less severe since a similarly low implementation have been reported in men’s professional football previously.^19^ One potential explanation for the relatively low usage of the NHE programme could be that the preventive efficacy of the programme has mainly been documented in subelite teams.^5–8^ Furthermore, just because a preventive measure has been proven efficacious in clinical trials it does not guarantee that it is also efficient in a real-life setting.^28 29^ It is thus encouraging that the elite teams that used the NHE in this present study reported fewer hamstring injuries and that a similarly good effect of the exercise was shown in elite male teams recently.^19^

A second potential explanation is that four out of the six teams that used the NHE in the team or individual training in the present study reported complaints (effectiviness) of muscle stiffness or soreness after training sessions. This fact, in combination with the tight match schedule of professional football teams, could have a negative effect on the use of the NHE due to fear that the exercise may have a negative effect on the player’s ability to perform during matches.

### How can we improve adoption and implementation of the NHE programme?

There is a good knowledge and a positive attitude to the NHE among medical staff in elite women’s football. However, for a preventive measure to be successful it is important that players, coaches and officials are motivated to use the programme.^30^ Lack of football specificity or the validity of the suggested preventive measures may cause concerns for coaches. This calls for further studies in elite settings, and above all in women football elite teams. As discussed in a previous study,^19^ it is important to get the coaches on board, improve the quality of the internal communication^31^ and to establish the minimum effective dose to improve the adherence.^9^

### Can we explain why teams that used the NHE in team training had a lower incidence of hamstring injuries at matches only?

The incidence of hamstring injuries at matches was significantly lower in the team group compared with the individual training group but not for the incidence of hamstrings injuries at training, a similar finding as in a previous male study.^19^ This difference is most likely explained by the difference in high-intensity actions between training and matches. Most teams have two matches a week during the competitive season, and the training sessions between matches are often focused on recovery.

## Strengths and limitations

A strength of the current study is that 100% of the included teams completed the questionnaire. The study is also strengthened by the homogeneity of the cohort, consisting only of professional European women’s football teams.

It should however be acknowledged that the study relies on a relatively small sample size and a short observation period, thus an exploratory nature of this work. A larger study sample and a longer study period would have increased the possibility to draw conclusions about potential associations between the use of the NHE programme and hamstring injury occurrences and reduce the risk of the results being affected by seasonal variation, detect dose-response effects and also improve the generalisability. The study is also limited by the fact that no detailed information about the included teams training content were available. We can thus not make any assumptions based on training principals and content, other than the inclusion of the NHE, which could potentially influence hamstring injury rates and that could potentially also differ between teams that included the NHE in their training and teams that did not. We should also acknowledge that individual players may perform supplementary training sessions in addition to their training sessions with their respective teams. Since the NHE is a relatively well-known exercise it is plausible that it could be included in potential supplementary training sessions and the use of NHE among individual players could thus potentially be larger than reported in this current study.

## Conclusions

An excellent reach, but a low adoption of the NHE programme was reported for women’s elite teams in Europe during the 2020–21 season. Teams that used NHE for the whole team or most players had a lower hamstring injury incidence at match-play than teams that did not use the NHE or used it for individual players only.

## Data Availability

No data are available. The coded dataset is confidential, and no data can be publicly available.
